# Comparative Xylose Metabolism among the Ascomycetes *C. albicans*, *S. stipitis* and *S. cerevisiae*

**DOI:** 10.1371/journal.pone.0080733

**Published:** 2013-11-13

**Authors:** Doreen Harcus, Daniel Dignard, Guylaine Lépine, Chris Askew, Martine Raymond, Malcolm Whiteway, Cunle Wu

**Affiliations:** 1 Biotechnology Research Institute, National Research Council, Montréal, Quebec, Canada; 2 Hôpital Maisonneuve-Rosemont, Montréal, Quebec, Canada; 3 Department of Biology, McGill University, Montréal, Quebec, Canada; 4 Institut de Recherche en Immunologie et en Cancérologie (IRIC), Université de Montréal, Montréal, Quebec, Canada; 5 Department of Biology, Concordia University, Montréal, Quebec, Canada; 6 Department of Medicine, Division of Experimental Medicine, McGill University, Montréal, Quebec, Canada; Institute of Biology Valrose, France

## Abstract

The ascomycetes *Candida albicans, Saccharomyces cerevisiae* and *Scheffersomyces stipitis* metabolize the pentose sugar xylose very differently. *S. cerevisiae* fails to grow on xylose, while *C. albicans* can grow, and *S. stipitis* can both grow and ferment xylose to ethanol. However, all three species contain highly similar genes that encode potential xylose reductases and xylitol dehydrogenases required to convert xylose to xylulose, and xylulose supports the growth of all three fungi. We have created *C. albicans* strains deleted for the xylose reductase gene *GRE3*, the xylitol dehydrogenase gene *XYL2*, as well as the *gre3 xyl2* double mutant. As expected, all the mutant strains cannot grow on xylose, while the single *gre3* mutant can grow on xylitol. The *gre3* and *xyl2* mutants are efficiently complemented by the *XYL1* and *XYL2* from *S. stipitis*. Intriguingly, the *S. cerevisiae GRE3* gene can complement the *Cagre3* mutant, while the *ScSOR1* gene can complement the *Caxyl2* mutant, showing that *S. cerevisiae* contains the enzymatic capacity for converting xylose to xylulose. In addition, the *gre3 xyl2* double mutant of *C. albicans* is effectively rescued by the xylose isomerase (XI) gene of either *Piromyces* or *Orpinomyces*, suggesting that the XI provides an alternative to the missing oxido-reductase functions in the mutant required for the xylose-xylulose conversion. Overall this work suggests that *C. albicans* strains engineered to lack essential steps for xylose metabolism can provide a platform for the analysis of xylose metabolism enzymes from a variety of species, and confirms that *S. cerevisiae* has the genetic potential to convert xylose to xylulose, although non-engineered strains cannot proliferate on xylose as the sole carbon source.

## Introduction

Sugars represent an important source of carbon and energy for fungi. However, different species can have widely differing capacities to use sugars for growth. In general, fungal cells prefer to use the 6-carbon sugar glucose, which they can convert to pyruvate and ultimately oxidize to CO_2_ and water with the concomitant production of up to 36 ATP units through the process of oxidative phosphorylation [1]. Because of this efficiency, many cells suppress the metabolism of other sugars in the presence of glucose [2]. However, in the absence of a direct glucose source other hexoses can be modified into glucose/fructose for entry into the glycolytic pathway, for example galactose can be modified to fructose through the action of the Leloir pathway enzymes [3,4]. As well, disaccharides and more complex sugars can be enzymatically converted to monosaccharides and ultimately to glucose/fructose for entry into the glycolytic pathway [5-7].

Pentose sugars are also a source of carbon and energy for fungi, but the metabolism of these sugars involves a distinct pathway relative to the hexose sugars. Cells make use of the transaldolase and transketolase reactions of the pentose phosphate pathway to generate 3 and 6 carbon intermediates from 5 carbon sugars [8,9]. The resulting hexose-phosphates and glyceraldehyde-3-phosphate enter into glycolysis and oxidative phosphorylation. 

Not all fungi make equivalent use of pentose sugars. As one of the most prevalent pentose sugars in nature, xylose makes up to 40% of hemicellulose and is highly abundant in the biosphere. Current efforts to replace fossil fuel with more renewable fuel supplies have led to interest in generating ethanol directly from biomass material; fermentation of xylose to ethanol is thus of commercial interest because it is such a large component of biomass feedstocks [9,10]. However, present production of commercial ethanol depends primarily on the fermentation of hexose sugars by *S. cerevisiae*. While this yeast ferments glucose to ethanol very efficiently, it is ineffective in metabolizing xylose [8,9]. Xylose fermenting yeasts such as *S. stipitis* are not as effective in industrial fermentation as *S. cerevisiae*, so efforts have been made to genetically modify *S. cerevisiae* by adding metabolic capacity from organisms that can efficiently ferment xylose [8,11]. 

Although *S. cerevisiae* is unable to metabolize xylose, it can utilize xylulose as a sole carbon source [12,13], suggesting that *S. cerevisiae* lacks the capacity of converting xylose to xylulose. Therefore genetic and metabolic engineering of *S. cerevisiae* in the past decades have involved transferring xylose reductase (XR) and xylitol dehydrogenase (XDH) genes, the two critical enzymatic activities for this conversion, from xylose fermenting organisms such as *S. stipitis*, but these efforts have generated only limited success [14]. The imbalance of co-factors caused by XR (requiring NADPH) and XDH (requiring NAD) in the process has been proposed to be an important issue, and efforts to overcome the problem have focused on identifying an XR that utilizes NADH instead NADPH [15]. An alternative approach has been to express fungal or bacterial xylose isomerases in *S. cerevisiae* to bypass the need of co-factors in the conversion of xylose to xylulose [16]. Both of these approaches have reported success in improving the ability of *S. cerevisiae* to metabolize xylose [11,16,17]. However, commercial application of these modified strains in the industrial fermentation of xylose to ethanol has not been achieved.

Comparative genomic studies show that both XR and XDH exist in *S. cerevisiae*, and they are well conserved when compared to other xylose metabolizing fungi such as *C. albicans* and *S. stipitis*. It is important to know if the *S. cerevisiae* XR and XDH have the expected enzymatic capacity, as this will help to design strategies to engineering *S. cerevisiae* for improved xylose metabolism. Here we show that XR and XDH of *S. cerevisiae* complement the corresponding function of these genes in the xylose metabolizing yeast *C. albicans*, demonstrating clearly that it is not the enzymatic activities per se, and may not even be the cofactor imbalance, that prevents an effective xylose metabolism in *S. cerevisiae*. Using *C. albicans* deletion mutants, we also demonstrate that xylose isomerases can bypass the requirement for XR and XDH to permit *C. albicans* growth on xylose as a sole carbon source. 

## Materials and Methods

### Media and culture conditions

Yeast strains in this study were grown in YPD or synthetic medium (0.67% Difco yeast nitrogen base without amino acids) supplemented with complete amino acids and filter sterilized D-glucose (2%), D-xylose (2%), xylitol (2%) or xylulose (0.5%) 

### Plasmid construction

Plasmids and oligonucleotides used in this study are listed in Tables 1 and 2. For complementation studies all genes were cloned into the integration plasmid CIpACT1-CYC, which contains the *Candida albicans ACT1* promoter, *CYC1* terminator and *RPS1* gene, to allow the cloned in gene of interest to be integrated at the *RPS1* locus [18] and expressed under the control of the *ACT1* promoter. Integration is targeted to *RPS1* by linearizing the plasmid with either a *Stu*I or *Nco*I digest. However a fraction of integrations were also found at *ACT1*. All plasmids used in this study were linearized with *Stu*I for integration with the exception of pDH271, which was linearized with *Nco*I. All constructs were sequenced to confirm integrity.

**Table 1:** Plasmids used in this study.

Plasmid	Description	Reference
pGEM-HIS1	pGEM-T (Promega) with *C. albicans* HIS1	[21]
pRSARG4Δ SpeI	pRS314 with *C. albicans* ARG4	[21]
pSN40	*pCR-BluntII- TOPO* with *C. maltosa* LEU2	[28]
pSFS2A	*C. albicans* SAT1 cassette	[22]
CIpACT-CYC	ACT1p-CYC1t-RPS1-cURA3	[18]
pDH270	CIpACT-CYC with *S. stipitis* XYL2	This study
pDH271	CIpACT-CYC with *S. stipitis* XYL1	This study
pDH275	CIpACT-CYC with *Piromyces* sp. XYLA	This study
pDH276	CIpACT-CYC with *Orpinomyces* sp. UKK1	This study
pDH277	CIpACT-CYC with *T. thermophilus* TTHXYLA (codon optimized for *Candida albicans*)	This study
pDH278	CIpACT-CYC with *C. cellulolyticum* xylose isomerase (codon optimized for *Candida albicans*)	This study
pDH279	CIpACT-CYC with *S. cerevisiae* SOR1	This study
pDH280	CIpACT-CYC with *S. cerevisiae* XYL2	This study
Plate 393	CIpACT-CYC with *S. cerevisiae* GRE3	This study
Plate 395	pUC57 with *S. cerevisiae* GRE3 (CTG/Leu to TTG)	This study
Plate 396	CIpACT-CYC with *S. cerevisiae* GRE3 (CTG/Leu to TTG)	This study
pGen5	pUC57 with *Piromyces* *sp.* XYLA	This study
pGen6	pUC57 with *Orpinomyces* *sp.* UKKI	This study
pGen7	pUC57 with *T. thermophilus* TTHXYLA (codon optimized for *Candida albicans*)	This study
pGen8	pUC57 with *C. cellulolyticum* xylose isomerase (codon optimized for *Candida albicans*)	This study

**Table 2:** Oligonucleotides used in this study.

Name	Sequence	Gene
MR2305	CAATTTTCTTTTTCGCCCTCGCACCACCCCTCCCCCTCCCTCTCCCTCCACACCTTCATATGTGGAATTGTGAGCGGATA	*CaGRE3*
MR2306	TAGTTTTTTTTTAATACGAGCCTACCACCACCAACTAATTTTTTTTTAGTCGATTTTGCGCAATTTTCTTTTTCGCCCTC	*CaGRE3*
MR2307	TATTTACATATTCAAAGATTTCAAAATATACACATTATATATATATATATATATATATGTGTTTTCCCAGTCACGACGTT	*CaGRE3*
MR2308	TATTAGGTAGACAAGCAGTTTTAGATGCCATTTAAAAAGAAAAAAAGAATGTTCACCTTTTATTTACATATTCAAAGATT	*CaGRE3*
MR2309	CCTTCCACATTCTTCAGATTGTTATGC	*CaGRE3*
MR2310	GGTGGTACCACTTCATTAGTTGATGC	*CaGRE3*
MR2311	CGTCTATAAATACACCTTGCAATCCCCCCCCCCATTCATTCGTTTTTTATTTCCCCAACCTGTGGAATTGTGAGCGGATA	*CaXYL2*
MR2312	TGATTTTTCGGAGAAATTGTTACTCCCAATTGTTACTCCTTATTTGTTGCTCCTAATTTTCGTCTATAAATACACCTTGC	*CaXYL2*
MR2313	GAAAAATTTTTCTTTTTTTTTATTCGACTATACACTTGGTAGCACTATAGATTTCTACATGTTTTCCCAGTCACGACGTT	*CaXYL2*
MR2314	GGGAGTGAGTTGGAAAGACATGGTTTGGTTGTTGTTTGGGGTGTGTCTTTTTTTTTTGGAGAAAAATTTTTCTTTTTTTT	*CaXYL2*
MR2315	GTAGACAGGAGAACTGGCTTACTCTG	*CaXYL2*
MR2316	GTGAGACAGATATTGTTGCCTGATGAG	*CaXYL2*
MR2511	CGTCTATAAATACACCTTGCAATCCCCCCCCCCATTCATTCGTTTTTTATTTCCCCAACCGAGCTCCACCGCGGTGGCGGCCGCT	*CaXYL2*
MR2512	GAAAAATTTTTCTTTTTTTTTATTCGACTATACACTTGGTAGCACTATAGATTTCTACATGGTACCGGGCCCCCCCTCGAGGAA	*CaXYL2*
MR2004	TAAATTTGTGTTGTTCGGTGACTCCATCAC	*SAT1*
MR2005	TCTCATATGAAAATTTCGGTGATCCCTGAG	*SAT1*
MR2357	CTGAAGAGTTCCCAACTTTGTCTGTTC	*LEU2*
MR2358	CCGTTACAGGAGTTAGTCATTCTTCAAC	*LEU2*
MR1322	GGAGTTCCATTTAGAGAAACTCATC	*ARG4*
MR1323	CGAAAATTCTCATATCGGTAGCAAC	*ARG4*
MR1324	GAAGTTGTGTGGGAAAAATCTTCAC	*HIS1*
MR1325	ACAGTTCACCTGGTACGGTTTTCTAG	*HIS1*
ODH322	TCCcccgggATGACTGCTAACCCTTCCTTGG ^a^	*SsXYL2*
ODH323	CTAGtctagaTTACTCAGGGCCGTCAATGAGA	*SsXYL2*
ODH326	TCCcccgggATGCCTTCTATTAAGTTGAACTCTGGTTA	*SsXYL1*
ODH328	CTAGtctagaTTAGACGAAGATAGGAATCTTGTCCCAGTCCCAAGGGTCGTTGAATCTCAAG	*SsXYL1*
ODH352	CTGCAGacgcgtATGTCTCAAAATAGTAACCCT	*ScSOR1*
ODH353	GGAATTgtcgacTCATTCAGGACCAAAGATAATAGT	*ScSOR1*
ODH354	CTGCAGacgcgtATGACTGACTTAACTACACAAGAA	*ScXYL2*
ODH355	GGAATTgtcgacTCATTCCGGGCCCTCAATGATCGT	*ScXYL2*
ODH346	TGATACCGACAGTATCATCGAT	*ACT1* flanking
p517	TTTCTGGTGAATGGGTCAACGAC	*RPS1*
p544	AATAGAGAGAAACTATATTATACAC	*RPS1* flanking
p565	TTTTCTAATTTTCACTCCTGG	*ACT1* promoter
p641	GCCTTCTATTAAGTTGAACTCTGG	*SsXYL1*
p642	ATGACTGCTAACCCTTCCTTGG	*SsXYL2*
p643	AATGAGACACTTGACAGCACCC	*SsXYL2*
p644	TCGTTGAATCTCAAGTTGATGTCC	*Ss*XYL1
p645	ACCCAATATCTAATTTAGAAATAGC	*CaGRE3*
p648	GAAAATTGGAAAacgcgtAAAGATGTCTTCACTGGTTACTC	*ScGRE3*
p649	GATTTTACTGgctagcTCAGGCAAAAGTGGGGAATTTACC	*ScGRE3*
p650	AATTTACCATCCAACCAGGTCC	*ScGRE3*
p657	AAATTGACAAAAAAGTCTGTGCG	*ScGRE3*
p658	TGTATAAATATCAAACGATTTCTCC	*CaGRE3*

a Lower case letters are restriction sites.

The open reading frames of the *S. stipitis* (*Ss*.) genes *XYL1* and *XYL2* were amplified by PCR from *Ss*. (CBS 6054) genomic DNA using primers ODH326/328 and ODH322/323 respectively. The products were cloned into the *Sma*I/*Nhe*I linearized vector CIpACT1-CYC to give rise to pDH271 and pDH270 respectively. ODH328 was designed to mutate the *Nco*I site in the *XYL1* gene so the plasmid could later be linearized with *Nco*I for integrating at *RPS1*. The *S. cerevisiae* (*Sc*.) *SOR1* and *XYL2* coding regions were PCR amplified from *S. cerevisiae* (BY4743) genomic DNA with ODH352/353 and ODH354/355 respectively and the products cloned to the *Mlu*I/*Sal*I sites of CIp-ACT1-CYC to produce pDH279 and pDH280. The coding region for the *ScGRE3* gene, which contains 5 CTG/Leu codons, was amplified by PCR from *S. cerevisiae* (BY4743) genomic DNA with primers p648/649 and cloned at the *Mlu*I/*Nhe*I sites of the vector CIpACT1-CYC to produce plasmid Plate 393. For the “candidized” version, the coding region for the *ScGRE3* gene, with the addition of flanking MluI and NheI restriction sites, was synthesized (GenScript, New Jersey) with each of the 5 CTG/Leu codons changed for TTG/Leu codons and the synthetic gene cloned in vector pUC57 to produce plasmid Plate 395. Plasmid Plate 395 was then digested with *Mlu*I and *Nhe*I; the 1-kb fragment for the “candidized” *ScGRE3* coding region was gel-purified and cloned in vector CIpACT1-CYC to produce plasmid Plate 396.

The open reading frames of 4 xylose isomerase genes were also synthesized by GenScript: *XYLA* from *Piromyces* sp. (CAB76571), *Orpinomyces* sp. (ACA65427) designated *Orp*XI, *TTHXYLA* from *Thermus thermophilus* (BAA14301) and *Clostridium cellulolyticum* (YP_002507697) designated as *Ccel*XI. *TTHXYLA* and *Ccel*XI were codon optimized for *Candida albicans*. The start codon for *TTHXYLA* was changed from GTG to ATG and *Sal*I and *Nhe*I sites were added 5’ and 3’ respectively. *Xho*I and *Spe*I sites were added 5’- and 3’-end respectively to *XYLA*, *Orp*XI and *Ccel*XI. The synthesized genes were supplied in the vector pUC57 at the *Eco*RV site. 

To clone these genes into the integrating plasmid, the *XYLA, Orp*XI and *Ccel*XI genes were each cut out from pUC57 with an *Xho*I digestion, blunt-ended, and subsequently digested with *Spe*I. These genes were then cloned into the CIpACT1-CYC plasmid at the *Sma*I/*Nhe*I sites to give rise to pDH275, pDH276 and pDH278 respectively. pDH277 was constructed similarly, except that the *TTHXYLA* gene was moved out of the pUC57-based construct with a *Sal*I digestion, blunt-ended and followed by an *Nhe*I digestion. Cloning in this way removed an out-of-frame ATG between the *Sma*I and *Sal*I site of the vector. 

### Strain construction and manipulation

All strains used in this study are listed in Table 3. All *C. albicans* strain constructions, transformations, and subsequent manipulations were carried out using standard procedures [19,20]. *Candida* deletion strains were all constructed in strain SN148 using PCR-based gene targeting [21]. To construct the *gre3-9* and *xyl2-16* deletion strains, two runs of PCR using 80-mers were performed on plasmids pGEM-*HIS1* or pRSARG4∆SpeI, and the PCR products were used to replace the entire targeted ORF of either *GRE3* or *XYL2* at one allele marked with *HIS1* and the second allele with *ARG4*. To construct the *Candida gre3/xyl2-3* double deletion mutant, *XYL2* was deleted in the *gre3-9* strain. PCR products generated from plasmids pSN40 or pSFS2A were used to replace the entire targeted ORF at one allele with *LEU2* and the second allele with *SAT1*. The SAT1-FLP cassette [22] was then looped out to leave behind only the FRT sequence. 

**Table 3:** Strains used in this study.

Strain	Genotype	Reference
CBS 6054	*Scheffersomyces stipitis* wild-type isolated from insect larvae	ATCC 58785
SC5314	*Candida albicans* wild-type clinical isolate	[37]
BY4741	MATa *his3*∆1 l*eu2*∆0 *met15*∆0 *ura3*∆0.	[38]
BY4743	*MAT*a/*MAT*alpha *his3*∆1/*his3*∆1 *leu2*∆0/*leu2*∆0 *lys2*∆0/*LYS2 MET15*/*met15*∆0 *ura3*∆0/*ura3*∆0(4741/4742)	[38]
SN148	*arg4*∆/*arg4*∆ *leu2*∆/*leu2*∆ *his1*∆/*his1*∆ *ura3*∆::imm434/*ura3*∆::imm434 *iro1*∆::imm434/*iro1*∆::imm434	[28]
*gre3-9*	SN148 *gre3*::*HIS1*/*gre3*::*ARG4*	This study
*xyl2-16*	SN148 *xyl2::HIS1*/*xyl2*::*ARG4*	This study
*gre3/xyl2-3*	*gre3::HIS1/gre3::ARG4 xyl2::LEU2/xyl2::FRT*	This study
CDH120	*gre3::HIS1/gre3::ARG4 RPS1/RPS1*:: [CIpACT-CYC - *S. stipitis XYL1* (pDH271)]	This study
CDH116	*xyl2::HIS1/xyl2::ARG4 RPS1/RPS1*:: [CIpACT-CYC - *S. stipitis XYL2* (pDH270)]	This study
CDH139	*xyl2::HIS1/xyl2::ARG4 RPS1/RPS1*:: [CIpACT-CYC- *S. cerevisiae SOR1* (pDH279)]	This study
CDH128	*gre3::HIS1/gre3::ARG4 xyl2::LEU2/xyl2*::FRT *RPS1/RPS1*:: [CIpACT-CYC *Piromyces* sp. *XYLA* (pDH275)]	This study
CDH129	*gre3::HIS1/gre3::ARG4 xyl2::LEU2/xyl2*::FRT *RPS1/RPS1*:: [CIpACT-CYC Orpinomyces sp. *UKK1* (pDH276)]	This study
CDH130	*gre3::HIS1/gre3::ARG4 xyl2::LEU2/xyl2*::FRT *RPS1/RPS1*:: [CIpACT-CYC *T. thermophilus TTHXYLA* (pDH277)]	This study
CDH131	*gre3::HIS1/gre3::ARG4 xyl2::LEU2/xyl2*::FRT *RPS1/RPS1*:: [CIpACT-CYC *C. cellulolyticum* xylose isomerase (pDH278)]	This study
CDH140	*gre3::HIS1/gre3::ARG4 RPS1/RPS1*:: [CIpACT-CYC - *Piromyces* sp. *XYLA* (pDH275)]	This study
CDH141	*xyl2::HIS1/xyl2::ARG4 RPS1/RPS1*:: [CIpACT-CYC - *Piromyces* *sp. XYLA* (pDH275)]	This study
CA220	*gre3::HIS1/gre3::ARG4 xyl2::LEU2/xyl2*::FRT *RPS1/RPS1*:: [CIpACT-CYC *S. stipitis XYL2* (pDH270)]	This study
CA227	*gre3::HIS1/gre3::ARG4 xyl2::LEU2/xyl2*::FRT *RPS1/RPS1*:: [CIpACT-CYC *S. stipitis XYL2* (pDH270)] ura3^-^/ura3^-^	This study
CA242	*gre3::HIS1/gre3::ARG4 xyl2::LEU2/xyl2*::FRT *RPS1/RPS1*:: [CIpACT-CYC *S. stipitis XYL2* (pDH270)]::[CIpACT-CYC *S. stipitis XYL1* (pDH271)]	This study
CA243	*gre3::HIS1/gre3::ARG4 xyl2::LEU2/xyl2*::FRT *RPS1/RPS1*:: [CIpACT-CYC *S. stipitis XYL2* (pDH270)]::[CIpACT-CYC *S. stipitis XYL1* (pDH271)]	This study

All strains used for complementation analyses were created by chromosomally integrating the desired genes at either the *RPS1* or the *ACT1* locus. Specifically, the *C. albicans gre3-9* strain was transformed with the plasmid pDH271 carrying the *SsXYL1* gene to create strain CDH120. Transformation of strain *xyl2-16* with pDH270 carrying *SsXYL2* gave rise to strain CDH116, and pDH279 carrying *ScSOR1* created the strain CDH139. The complementation of the *Candida gre3/xyl2-3* double deletion strain was carried out with the *S. stipitis XYL1* and *XYL2* homologs. The *xyl2* complementation was done first with plasmid pDH270 linearized with *Stu*I, and integration at *RPS1* was confirmed by PCR (with primers p565/p544). The resulting strain, named CA220, was grown in liquid synthetic medium with xylitol, then selected on a 5-fluoroorotic acid (5-FOA) plate containing xylitol as the sole carbon source to “recycle” the *URA* auxotrophic marker and retain the xylitol dehydrogenase function to give rise to the *ura* ^-^ strain CA227. Transformation of CA227 with *Nco*I linearized pDH271 (*SsXYL1*) followed by selection on 2% xylitol-uridine plates gave rise to strains CA242 and CA243. 

Complementation of the *gre3-9* strain with *ScGRE3* was carried out with *Stu*I linearized Plate 393 to produce strain CA247, and with *Stu*I linearized Plate 396 to produce strains CA255 and CA256. Transformation of the *gre3-9*/*xyl2-16* double deletion strain with pDH275, pDH276, pDH277 or pDH278 constructs harboring the heterologous xylose isomerases created strains CDH128 (*Piromyces*), CDH129 (*Orpinomyces*), CDH130 (*Thermus thermophilus*) and CDH131 (*Clostridium cellulolyticum*) respectively. The single deletion strains *gre3-9* and *xyl2-16* were also transformed with the *Piromyces* isomerase plasmid pDH275 to create strains CDH140 and CDH141.

### Growth curves

Overnight cell cultures were grown in synthetic complete medium containing dextrose (2%). Cells were washed once with sterile water before diluting to an OD_600nm_ of 0.2 in 5-10 ml of synthetic complete medium containing xylose (2%), xylitol (2%) or xylulose (0.5%). Cells were grown aerobically at 30°C with shaking, and the OD_600nm_ was taken at intervals for up to two weeks.

### Microarrays analysis

The strain BY4743 of *S. cerevisiae* was used for transcription profiling in xylose medium. A 10-ml starter culture in 2% raffinose (to minimize glucose repression effect) was grown for about 2 days and used to inoculate a 400-ml culture in 2% raffinose. This culture was grown to an OD_600_ of approximately 0.75 - 0.8 (24-34 hr). The cells were then washed twice with sterile water. The cell pellet was resuspended in 8 ml of synthetic medium without sugar (S), and 2 ml was used to inoculate 100 ml cultures, either without sugar (S), with xylose (SX) or with dextrose (SD) for the reference. These cultures were incubated until an increase in OD_600_ was observed for the culture with dextrose (1.5 - 2 hr); there was no increase in the OD_600_ for the culture in xylose medium. 

The *Candida albicans* strain SC5314 was used for microarray in xylose medium.

Starter cultures in xylose and in dextrose media were grown overnight and used to inoculate the xylose and dextrose subcultures, respectively, and these were grown to an OD_600_ of approximately 0.8.

Cells were disrupted with glass beads in the FastPrep-24 instrument (MP Biomedicals). RNA was extracted with the RNeasy kit (QIAGEN cat. 74104), and 20 µg of total RNA was used for the cDNA synthesis with either cyanine-3 or cyanine-5 dyes. Probe synthesis, microarray hybridizations, washings and analyses were as described [23,24]. *S. cerevisiae* microarrays (Y6.4k7, 6240 ORFs) were obtained from the University Health Network Microarray Centre (http://www.microarrays.ca, Toronto, Canada). *C. albicans* microarrays (double-spotted 6,394 intragenic 70-mer oligonucleotides) were obtained from the Biotechnology Research Institute microarray facility center (http://www.nrc cnrc.gc.ca/eng/services/bri/microarray.html, Montreal, Canada). The data sets for these array experiments are provided as excel files as supplementary S2, and the full data sets have been registered at NCBI with the GEO accession number GSE50476.

## Results

### *S. cerevisiae*, *S.* stipitis and *C. albicans* share conserved xylose metabolic enzymes but have different capacities for metabolizing xylose

The baker’s or brewer’s yeast *S. cerevisiae*, the fungal pathogen *C. albicans*, and the pentose-sugar fermenting yeast *S. stipitis* (formerly *Pichia stipitis*) are related ascomycetes, with *C. albicans* and *S. stipitis* in a lineage that uses the codon CTG to encode serine, unlike *S. cerevisiae*, which, consistent with the universal genetic code, uses CTG to encode leucine. These ascomycetes have distinct ecological niches; *S. cerevisiae* is almost a domesticated organism primarily associated with human brewing and baking, *C. albicans* is a human commensal colonizing mucosal surfaces and the gastrointestinal system, while *S. stipitis* inhabits the guts of some insects. These niches all provide efficient access to the hexose sugar glucose, but only the guts of wood-eating insects provide high levels of pentose sugars such as xylose for possible metabolism. 

**Figure 1:**
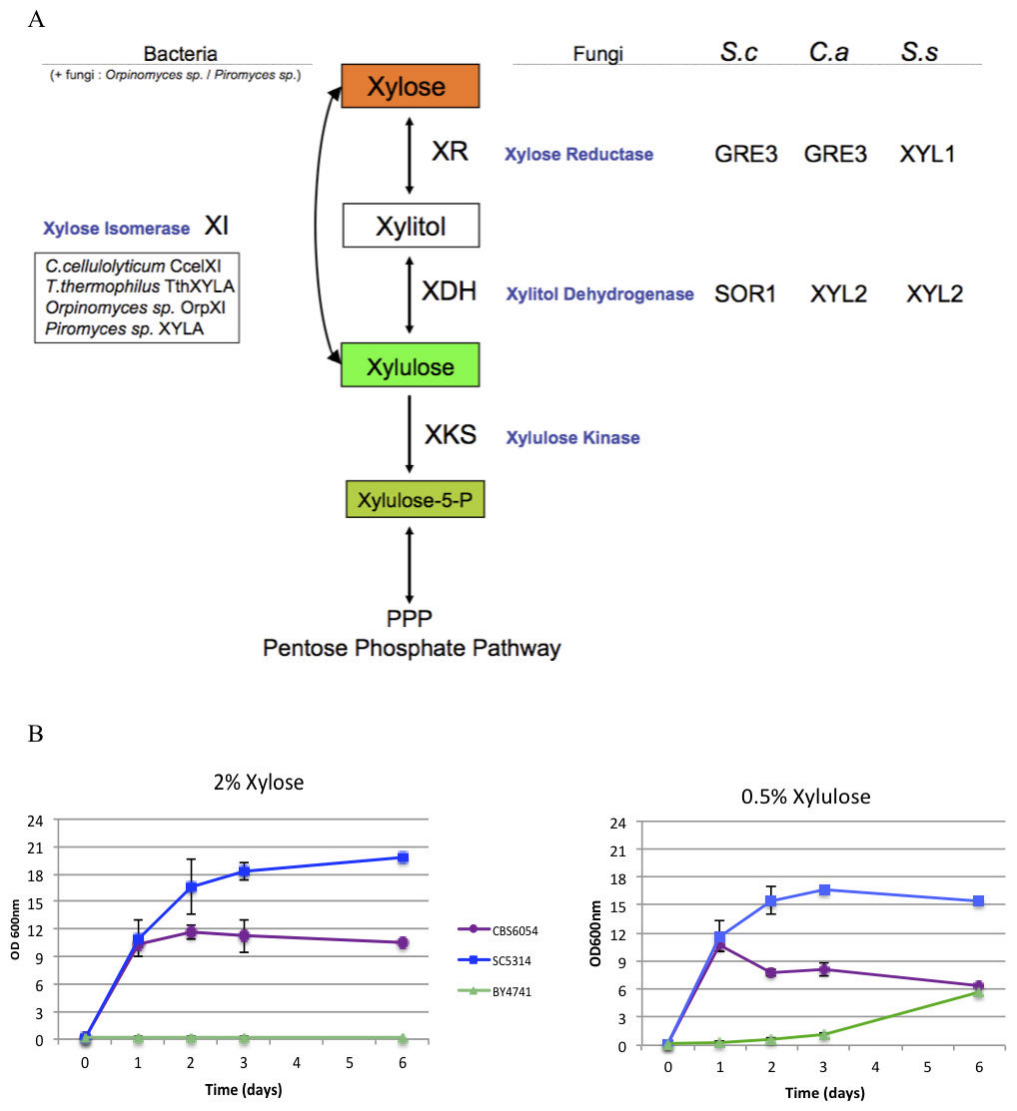
Xylose entry to the PPP pathway and xylose metabolizing capacities of different ascomycetes. A) The schematic presentation of conserved genes encoding enzymatic activities for the xylose entry into the PPP pathway in ascomycetes. *C*. *a* for *Candida albicans*, *S.* *c* for *Saccharomyces cerevisiae*, and *S*. s for *Schefferomyces stipitis*. B) Growth of *C. albicans* (SC5314)*, S. stipitis* (CBS6054) and *S. cerevisiae* (BY4741) in SC with 2% xylose or 0.5% xylulose. Strains were grown aerobically at 30°C and the optical density measured over a period of six days, n=3.

Although each of these organisms has different access to xylose as a potential carbon source, they all encode the enzymatic capacity to direct xylose into the pentose phosphate pathway. In fungi in general, the metabolism of xylose involves its transformation to xylulose through a two-step process, the first the reduction of xylose to xylitol by the action of a xylose reductase (XR), and the second the production of xylulose from xylitol by the action of a xylitol dehydrogenase (XDH) (Figure 1A). Sequence analysis indicates that all three species have genes that are predicted to encode these functions, and these genes are conserved (Figure S1). The *GRE3* genes of *C. albicans* and *S. cerevisiae*, and the *XYL1* gene of *S. stipitis*, are highly related sequences encoding predicted xylose reductase activity. The *XYL2* genes of *S. stipitis* and *C. albicans*, as well as the *XYL2*, *SOR1* and *SOR2* genes of *S. cerevisiae* are also closely related genes encoding putative dehydrogenases. The *SOR1* and *SOR2* genes, although annotated as sorbitol dehydrogenases, are more similar to the *C. albicans* and *S. stipitis XYL2* genes than is the yeast gene designated *XYL2*. Intriguingly however, although the species have similar genomic potential for xylose utilization, they actually have very different growth behaviors on this sugar. As shown in Figure 1B, while strains of each organism were able to grow in medium containing xylulose as the sole carbon source, the *S. cerevisiae* strain was not able to grow when the carbon source was the related pentose sugar xylose. Overall, *Candida* grows better than the others in both xylose and xylulose, while *S. cerevisiae* grows slowly in xylulose, and *S. stipitis* grows well in both xylose and xylulose, but reaches a plateau after 1 day of incubation in both sugars. 

### Transcription profiling of cellular response to xylose

**Figure 2:**
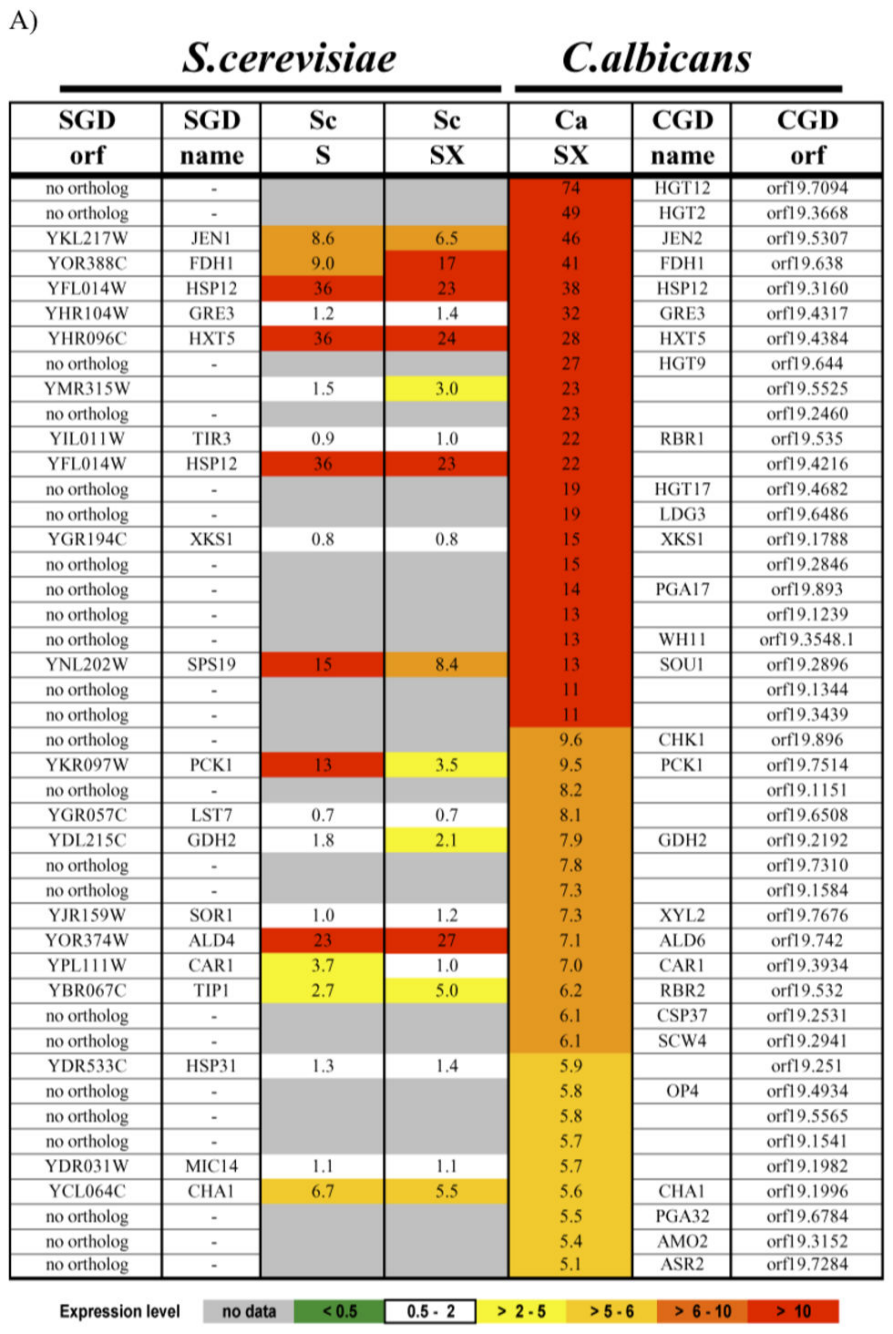
Transcription profile of *C. albicans* and *S. cerevisiae* in xylose A) From microarray experiments with *C. albicans* grown in xylose, 175 genes have at least a 2-fold transcription increase in xylose when compared to cells in dextrose. The 44 genes with more than a 5-fold induction in xylose (SX) are shown with their corresponding value. Data for *S. cerevisiae* is shown for comparison, and in gray-shade if no reciprocal best-hit ortholog is found. B) Transcription modulation of the *S. cerevisiae* sugar transporters in xylose. The data from the microarray experiments in xylose (SX) and in no-sugar condition (S) is shown for 18 members of the sugar transporter family, relative to cells in dextrose. *HXT5* and *HXT2* are likely induced due to the absence of dextrose (glucose). *HXT6*, *HXT7* and *HXT4* are induced specifically by the presence of xylose. *HXT4*, *HXT1* and *HXT3* show a transcriptional reduction only for the no-sugar condition (S), likely due to the absence of sugar. A summary for the transporters description from SGD is reported in the ‘SGD description’ column.

Previous work has established that in response to growth in xylose, *S. stipitis* induces the genes for XR and XDH, as well as for some sugar transporters [25,26]. We have examined the response of both *C. albicans* and *S. cerevisiae* to incubation with xylose as the sole carbon source. As shown in Figure 2A, C *. albicans* generates a robust signal when shifted from growth in glucose to xylose. 175 genes have at least a 2-fold transcription increase in xylose when compared to cells in glucose, of which 44 genes with more than a 5-fold induction in xylose are listed in the table. The induced genes include those that are critical for xylose metabolism such as *GRE3* (30 fold) and *XYL2* (7 fold), as well as the xylulose kinase *XKS1* (5 fold). Some genes encoding sugar transporters, including *HGT2, HGT12*, *HXT5*, *HGT9* and *HGT17*, are also strongly induced. This shows that *C. albicans* is capable of shifting its metabolic constitution to exploit xylose as a carbon source. In contrast, *S. cerevisiae* has no cell growth under these conditions, has a very modest response to the shift from glucose to xylose. This response is quite similar to that seen when shifting yeast cells from glucose medium to a medium containing no sugar source (Figure 2B). This behavior is consistent with the inability of *S. cerevisiae* to grow on xylose as the sole carbon source, although *S. cerevisiae* is capable of transporting xylose [27]. The data sets for the *C. albicans* and *S. cerevisiae* transcriptional profiling results are presented as Figure S2.

### GRE3 and XYL2 are necessary for xylose metabolism in *C. albicans*

To examine whether *GRE3* and *XYL2*, encoding potential xylose reductase (XR) and xylitol dehydrogenase (XDH) activities respectively, are solely responsible for the metabolism of xylose in *C. albicans*, we created disruption mutants of both genes. The two alleles of either *GRE3 or XYL2* were replaced by *HIS1* and *ARG4* in strain SN148 [28]. A double mutant, *gre3 xyl2*, was created by replacing the two alleles of *XYL2* with the *LEU2* and *SAT1* markers in a strain already deleted for *GRE3*. All the resulting strains were unable to grow on xylose as a sole carbon source, while as expected the *gre3* mutant strain was able to grow on xylitol (Figure S3). This confirms that in *C. albicans* the oxido-reductase activities encoded by the *GRE3* and *XYL2* genes are solely necessary for the metabolism of xylose.

### Complementation of gre3 and xyl2 mutants

**Figure 3:**
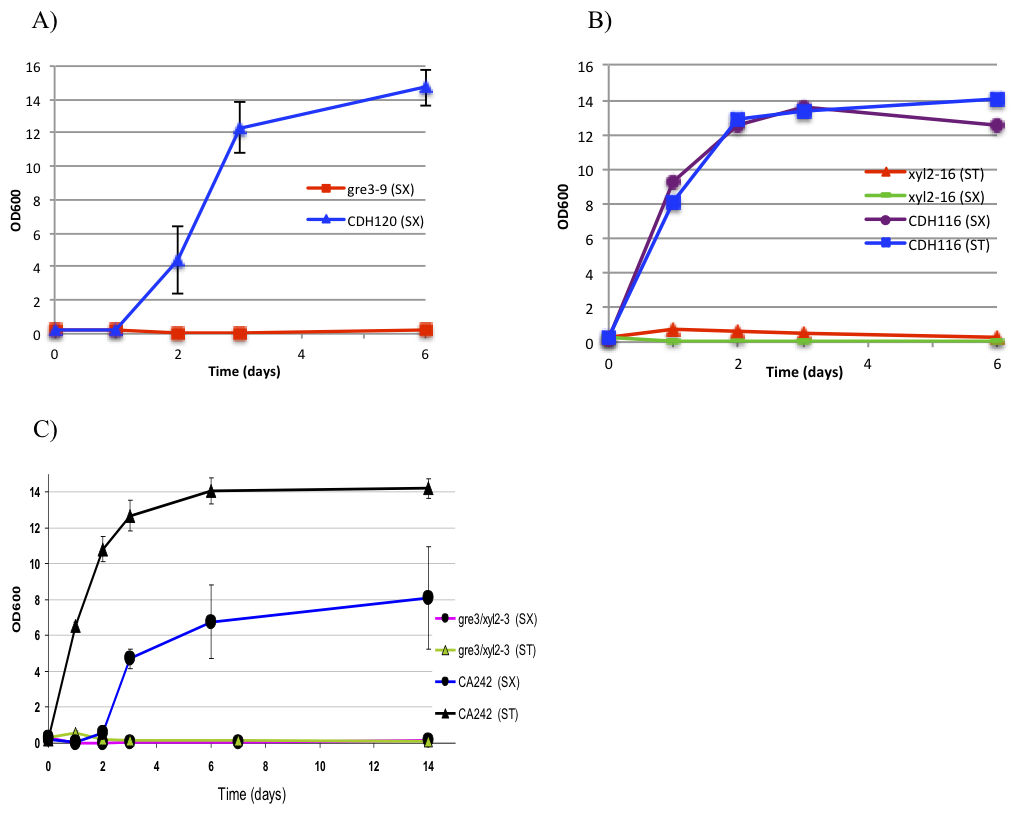
The xylose reductase and xylitol dehydrogenase genes from *S. stipitis* complement *C. albicans* deletion mutants of the equivalent genes. (A) *S. stipitis* *XYL1* (CDH120), and (B) *S. stipitis* *XYL2* (CDH116) complement the *C. albicans* *gre3* and *xyl2* deletion mutants respectively. (C) The *S. stipitis* *XYL1-XYL2* module (CA242) complements the *C. albicans* *gre3* *xyl2* double deletion mutant. Strains were grown aerobically at 30°C in SC with 2% xylose (SX) or 2% xylitol (ST). The optical density was measured over a period of up to 14 days, n=3.

We asked whether the corresponding XR and XDH genes of *S. stipitis* were capable of complementing the *C. albicans gre3* and *xyl2* deletions. Because both *C. albicans* and *S. stipitis* belong to the CUG clade, there was no need to modify the *S. stipitis* genes prior to introducing them into the *C. albicans* mutants. The *S. stipitis XYL1* gene integrated at the *RPS1* locus of *C. albicans* was capable of supporting robust growth of the *gre3* strain within two to three days in xylose medium (Figure 3A). As well, integration of the *S. stipitis XYL2* gene in the *C. albicans xyl2* strain complemented the deletion strains growth defect within one day in both xylitol or xylose medium (Figure 3B). We also tested the *S. stipitis* XR-XDH modular unit in the *Candida gre3/xyl2* double deletion mutant in xylose metabolism. Integration of *S. stipitis XYL1* and *XYL2* into the *gre3/xyl2* strain allowed the transformed *Candida* mutant to grow in medium using xylose as the sole carbon source, indicating that the XR-XDH module from *S. stipitis* is functional in *C. albicans* (Figure 3C). However, we noticed that these *Candida* cells showed a lag time of approximately 48 h when grown in medium with xylose compared to medium with xylitol as a carbon source. This lag was not observed with wild-type *C. albicans* cells, suggesting that the *S. stipitis* XR-XDH module may not be perfectly coupled to the other parts of the xylose metabolic pathway of *C. albicans*.

**Figure 4:**
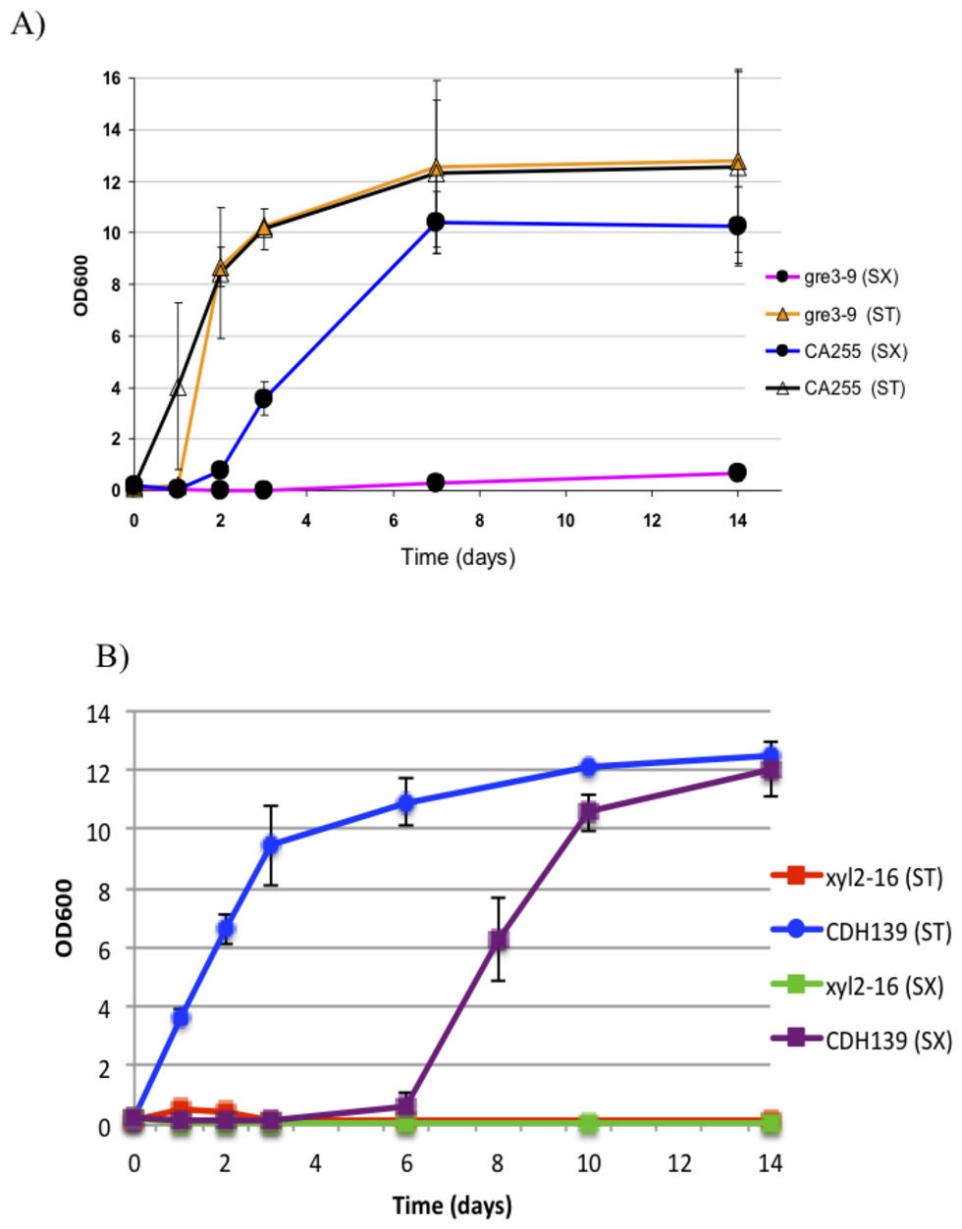
The xylose reductase and xylitol dehydrogenase genes from *S. cerevisiae* complement *C. albicans* deletion mutants of the equivalent genes. (A) *S. cerevisiae* *GRE3* (CA255), and (B) *S. cerevisiae* *SOR1* (CDH139) complement the *C. albicans* *gre3* and *xyl2* deletion mutants respectively. Strains were grown aerobically at 30°C in 2% xylose (SX) or 2% xylitol (ST). The optical density was measured over a period of 14 days, n=3.

Similarly, we investigated the ability of the *S. cerevisiae* homologs to complement the *C. albicans* mutants. Because *ScGRE3* contained 5 CTG codons that would encode leucine in *S. cerevisiae* but serine in *C. albicans* we used a synthetic gene with the 5 CTG codons switched to TTG to assess complementation. As shown in Figure 4A, integration of the candidized *ScGRE3* gene at *RPS1* [18] could complement the *gre3* mutation. The CTG codon unmodified *ScGRE3* gene was not able to complement when introduced at the same site (data not shown), suggesting the change of one or more leucine residues to serines compromised the enzymatic function. We also assessed the ability of *SOR1* and *ScXYL2* to complement the *xyl2* mutant of *C. albicans*. The *SOR1* gene contained no CTG codons, and when integrated at *RPS1* was capable of complementing the *xyl2* mutant for growth on xylitol within one day, however complementation of growth in xylose took several days (Figure 4B) The *ScXYL2* gene contained 4 CTG codons, and was not capable of complementing the function (data not shown). This could represent problems caused by the leucine/serine discrepancy, or could simply reflect the point that the *SOR1* gene is more similar to the *C. albicans* gene than is *ScXYL2*. Nonetheless, the observation that *ScGRE3* and *SOR1* can complement the *Candida gre3* and *xyl2* mutants shows that *S. cerevisiae* has XR and XDH enzymatic capacity for the crucial xylose-xylulose conversion steps of xylose metabolism. 

When we tested XR-XDH as a modular unit from *S. cerevisiae* for its capacity to complement the *Candida gre3/xyl2* double deletion mutant in xylose. We found the *S. cerevisiae* XR-XDH module (*GRE3-SOR1*) showed a clear, but relatively weak ability to support the growth of the *C. albicans* double mutant on xylose medium. Significant cell growth takes about two weeks (data not shown), which is much slower growth compared to the more rapid growth conferred by either *ScGRE3* or *ScSOR1* when complementing a *gre3* or *xyl2* single mutation of *C. albicans*. These results, along with those from *S. stipitis*, may suggest that the proper coupling of all the steps in the metabolic pathway is as important as the enzyme activities *per se* in each step. 

### Fungal xylose isomerases enable a *Candida* gre3/xyl2 double mutant to metabolize xylose

**Figure 5:**
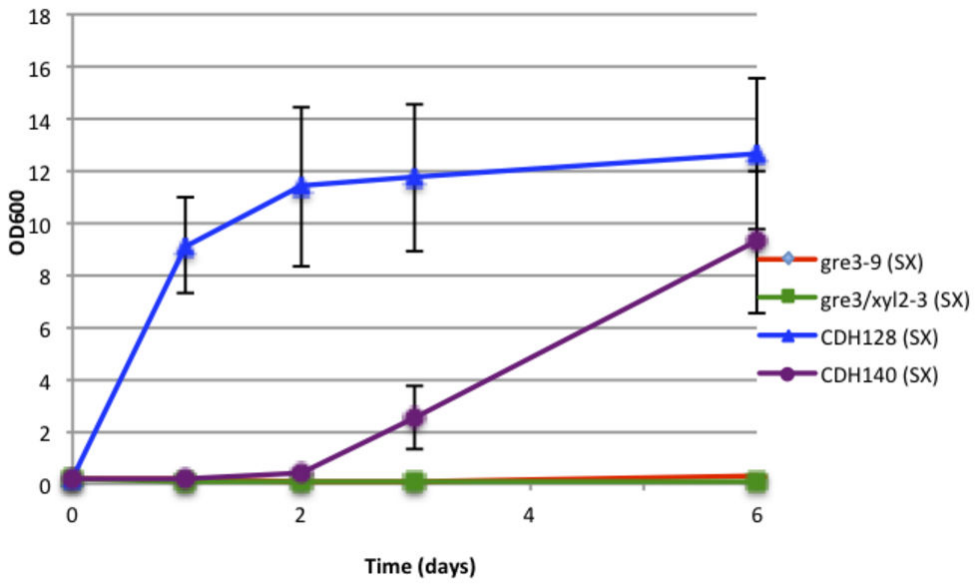
The xylose isomerase gene *XYLA* from *Piromyces* *sp.* complements a double *gre3/xyl2* deletion or single *gre3* deletion in *C. albicans*. Growth analysis in synthetic medium (SC) with 2% xylose as the sole carbon source for *C. albicans* strains: *gre3-9* (*gre3* deletion), *gre3/xyl2-3* (*gre3* *xyl2* double deletion), CDH128 (*gre3/xyl2-3* with the integrated *Pir*.*XYLA*) and CDH140 (*gre3-9* with the integrated *Pir*. *XYLA*). The optical density was measured over a period of 6 days, n=3.

We further assessed whether alternate enzymatic activities could function in the xylose utilization pathway of *C. albicans*. Because the role of XR and XDH is to transform xylose to xylulose, we asked whether expression of a heterologous xylose isomerase could complement the *gre3 xyl2* double mutant. Xylose isomerases exist in bacteria, in plants, as well as in some fungi, however, ascomycete fungi like yeast and *C. albicans* do not have endogenous xylose isomerases. Sequence analysis with phylogenic clustering of 17 xylose isomerases indicate that there are two classes of this enzyme, one represented by the enzyme from Piromyces, and the other from that of *Thermus thermophilus* as shown in the alignment (Figure S4). Genes corresponding to the XI from fungi (*Piromyces* *sp.* and *Orpinomyces* *sp.*), and bacteria (*Thermus thermophilus* and *Clostridium cellulolyticum*) were synthesized and introduced into the *gre3 xyl2* double mutant strain. *Piromyces* and *Orpinomyces* have the same codon usage as *C. albicans*, and thus required no CUG codon modification. *Thermus* and *Clostridium* have standard codon usage, so the xylose isomerase genes from these organisms were synthesized with CUG codon modification to reflect *C. albicans* codon usage. As shown in Figure 5, the isomerase from *Piromyces* allowed growth of the double mutant in xylose medium within one to two days indicating that the activity of the XI provides effective xylose-xylulose conversion. The same effect was observed with the *Orpinomyces* XI (data not shown). On the other hand the bacterial isomerases were not able to support the growth of the double the mutant in xylose medium; this could represent a problem with protein production, although the genes were codon optimized for expression in *C. albicans*, or a problem in enzyme function in situ. The *Piromyces* isomerase was also able to allow growth of the *C. albicans gre3* single deletion mutant in 3-4 days (Figure 5); however, it could not complement the growth of the *xyl2* single deletion mutant under the same conditions (data not shown), suggesting an inhibiting effect of accumulating xylitol on XI in *xyl2* mutant. 

## Discussion

Recent studies [26,29,30] have emphasized the genomic similarities of xylose utilizing fungi such as *C. albicans* and *S. stipitis*, and the non-utilizing yeast *S. cerevisiae*. Here we have extended these studies to show that when put in the common context of the *C. albicans* cell, genes for xylose metabolism from both the xylose metabolizing yeast *S. stipitis* and the non-utilizing yeast *S. cerevisiae* are able to complement the deletion of these functions and permit the growth of mutant *C. albicans* strains on xylose medium. In the correct context, the *GRE3* and *SOR1* genes of *S. cerevisiae* are capable of providing xylose reductase and xylitol dehydrogenase functions and of permitting cell growth on xylose as a sole carbon source. This emphasizes that it is not fundamentally the lack of xylose reductase and xylitol dehydrogenase capacity that prevents yeast cells from growing on xylose, but rather the regulatory and metabolic context of these enzymes.

A significant difference between *S. cerevisiae* and the xylose-utilizing yeasts *C. albicans* and *S. stipitis* is readily apparent when the organisms are examined for their transcriptional response to the sugar. Both *C. albicans* (Figure 2A; [26]) and *S. stipitis* [25,31] show robust changes in gene expression in response to the addition of xylose as a carbon source, and many of the induced genes encode components implicated in the transport or metabolism of the sugar. By contrast, as seen in Figure 2A and 2B the response of *S. cerevisiae* to the presence of xylose is very similar to the response to the absence of any sugar. However, the sugar transporter encoding genes *HXT4*, *HXT6* and *HXT7* have a modest induction that was unique to the xylose conditions, suggesting the cells may have been able to distinguish the presence of xylose from the absence of any sugar, even though the cells were not able to use the xylose for growth and proliferation. 

It has been proposed that the NADPH/NADH co-factor imbalance created by xylose reductase (XR) and xylitol dehydrogenase (XDH) plays a major role in preventing *S. cerevisiae* from utilizing xylose. Efforts have been made to change the XR preference for co-factors to alleviate the imbalance and improve xylose consumption [8,32-34]. However, the co-factor imbalance created by the enzymatic activities of XR and XDH with different co-factor preferences appears to be well resolved in *C. albicans*, suggesting that xylose-metabolizing fungi have the means to deal with the imbalance under aerobic conditions. We do not presently know what activities and processes are responsible for the phenotype. Intriguingly, many genes that are found to be unique to xylose utilizing yeasts are involved in NAD biosynthesis [26], and thus it would be interesting to see if any of these genes could improve xylose metabolism in *S. cerevisiae*. 

We have demonstrated that the sole xylose metabolic pathway in *C. albicans* is governed by the enzymatic activities encoded by *GRE3* and *XYL2*. Either single deletions or double deletion mutants are unable to metabolize xylose and therefore provide reliable tools and means to analyze the functionality of heterologous components of xylose metabolic pathways. We have analyzed the ability of *S. stipitis XYL1* and *XYL2* to complement the *C. albicans gre3 xyl2* mutant. While the *SsXYL1* and *XYL2* complement the *gre3 xyl2* in general, growth curve analyses indicate that the transformants grow better in xylitol than in xylose, and there is a 48-hour growth lag time in xylose that is absent in xylitol. However, we did not observe this in the single mutant complementation analyses. This suggests that the conversion of xylose to xylitol catalyzed by the Xyl1 reductase activity may be a limiting step, and the lag time likely reflects the time required for the initial accumulation of a critical amount of xylitol to be utilized as substrate for XDH, pointing to a direction of genetic and metabolic engineering to improve the step of xylose conversion. We have also analyzed four xylose isomerases for their ability to complement the *gre3 xyl2* double mutant, and found that the two fungal isomerases (from *Piromyces* and *Orpinomyces*) could complement the mutant *Candida*, whereas the two bacterial xylose isomerases (from *Thermus* and *Clostridium*) even when codon optimized for *Candida*, could not. The *Thermus thermophilus* XI activity, albeit at a low level, has been reported in *S. cerevisiae* [35], while the *Clostidium phytofermentans* (not *cellulolyticum*) enzyme was expressed in *S. cerevisiae* [36]. Presently, the molecular basis for this discrepancy in terms of functionality in *Candida* between the fungal and bacterial xylose isomerases remains unclear. Nevertheless, *Candida* mutants created in this study, combined with mutagenesis, could be powerful tools to evolve and identify desired enzymatic activities for xylose to xylulose conversion. This may help to engineer the yeast *S. cerevisiae* with a xylose isomerase function suitable for more robust performance in xylose metabolism.

## Supporting Information

Figure S1 **Alignments of A) xylose reductases and B) xylose dehydrogenases of *C. albicans*, *S.* stipitis and *S. cerevisiae*.**
(DOCX)

Figure S2 **Microarray data.** The transcription profiles for *S. cerevisiae* and *C. albicans*, when shifted to xylose from a glucose starter culture, were determined with two-color cDNA probe hybridizations. The tables show the average signal ratio (xylose/glucose, or no-sugar/glucose) of the replicates with the corresponding P-value for each ORF present on the microarrays. The complete data with the experimental details is available at NCBI under GEO accession number GSE50476.
(XLS)

Figure S3 **Growth analysis of *C. albicans* wild type (SC5314) and deletion mutants for xylose reductase (gre3-9) and xylitol dehydrogenase (xyl2-16) in synthetic medium (SC) with 2% xylose (SX) or 2% xylitol (ST).** Strains were grown aerobically at 30°C, and the optical densities were measured over a period of 4 days.
(DOCX)

Figure S4 **Alignment of fungal and bacterial xylose isomerases.**
(DOCX)

## References

[1] Kay J, Weitzman PDJ (1987) Krebs' citric acid cycle : half a century and still turning Kay J Weitzman PDJ London: London Biochemical Society.

[2] Trumbly RJ (1992) Glucose repression in the yeast Saccharomyces cerevisiae. Mol Microbiol 6: 15-21. doi:10.1111/j.1365-2958.1992.tb00832.x. PubMed: 1310793. 1310793

[3] Carlson M (1999) Glucose repression in yeast. Curr Opin Microbiol 2: 202-207. doi:10.1016/S1369-5274(99)80035-6. PubMed: 10322167. 10322167

[4] Leloir LF (1951) The enzymatic transformation of uridine diphosphate glucose into a galactose derivative. Arch Biochem Biophys 33: 186-190. doi:10.1016/0003-9861(51)90096-3. PubMed: 14885999. 14885999

[5] Ha SJ, Galazka JM, Kim SR, Choi JH, Yang X et al. (2011) Engineered Saccharomyces cerevisiae capable of simultaneous cellobiose and xylose fermentation. Proc Natl Acad Sci U S A 108: 504-509. doi:10.1073/pnas.1010456108. PubMed: 21187422. 21187422 PMC3021080

[6] Saitoh S, Tanaka T, Kondo A (2011) Co-fermentation of cellulose/xylan using engineered industrial yeast strain OC-2 displaying both beta-glucosidase and beta-xylosidase. Appl Microbiol Biotechnol 91: 1553-1559. doi:10.1007/s00253-011-3357-5. PubMed: 21643701. 21643701

[7] van Maris AJ, Abbott DA, Bellissimi E, van den Brink J, Kuyper M et al. (2006) Alcoholic fermentation of carbon sources in biomass hydrolysates by Saccharomyces cerevisiae: current status. Antonie Van Leeuwenhoek 90: 391-418. doi:10.1007/s10482-006-9085-7. PubMed: 17033882. 17033882

[8] Hahn-Hägerdal B, Karhumaa K, Jeppsson M, Gorwa-Grauslund MF (2007) Metabolic engineering for pentose utilization in Saccharomyces cerevisiae. Adv Biochem Eng/Biotechnol 108: 147-177. doi:10.1007/10_2007_062. PubMed: 17846723. 17846723

[9] Jeffries TW (2006) Engineering yeasts for xylose metabolism. Curr Opin Biotechnol 17: 320-326. doi:10.1016/j.copbio.2006.05.008. PubMed: 16713243. 16713243

[10] Saha BC (2003) Hemicellulose bioconversion. J Ind Microbiol Biotechnol 30: 279-291. doi:10.1007/s10295-003-0049-x. PubMed: 12698321. 12698321

[11] Van Vleet JH, Jeffries TW (2009) Yeast metabolic engineering for hemicellulosic ethanol production. Curr Opin Biotechnol 20: 300-306. doi:10.1016/j.copbio.2009.06.001. PubMed: 19545992. 19545992

[12] Bera AK, Ho NW, Khan A, Sedlak M (2011) A genetic overhaul of Saccharomyces cerevisiae 424A(LNH-ST) to improve xylose fermentation. J Ind Microbiol Biotechnol 38: 617-626. doi:10.1007/s10295-010-0806-6. PubMed: 20714780. 20714780

[13] Deng XX, Ho NW (1990) Xylulokinase activity in various yeasts including Saccharomyces cerevisiae containing the cloned xylulokinase gene. Scientific note. Appl Biochem Biotechnol 24-25: 193-199. doi:10.1007/BF02920245. PubMed: 2162148. 2162148

[14] Laluce C, Schenberg AC, Gallardo JC, Coradello LF, Pombeiro-Sponchiado SR (2012) Advances and Developments in Strategies to Improve Strains of Saccharomyces cerevisiae and Processes to Obtain the Lignocellulosic Ethanol-A Review. Appl Biochem Biotechnol 166: 1908-1926. doi:10.1007/s12010-012-9619-6. PubMed: 22391693. 22391693

[15] Verho R, Londesborough J, Penttilä M, Richard P (2003) Engineering redox cofactor regeneration for improved pentose fermentation in Saccharomyces cerevisiae. Appl Environ Microbiol 69: 5892-5897. doi:10.1128/AEM.69.10.5892-5897.2003. PubMed: 14532041. 14532041 PMC201209

[16] van Maris AJ, Winkler AA, Kuyper M, de Laat WT, van Dijken JP et al. (2007) Development of efficient xylose fermentation in Saccharomyces cerevisiae: xylose isomerase as a key component. Adv Biochem Eng/Biotechnol 108: 179-204. doi:10.1007/10_2007_057. PubMed: 17846724. 17846724

[17] Bettiga M, Bengtsson O, Hahn-Hägerdal B, Gorwa-Grauslund MF (2009) Arabinose and xylose fermentation by recombinant Saccharomyces cerevisiae expressing a fungal pentose utilization pathway. Microb Cell Factories 8: 40. doi:10.1186/1475-2859-8-40. PubMed: 19630951. PMC2720912 19630951

[18] Tripathi G, Wiltshire C, Macaskill S, Tournu H, Budge S et al. (2002) Gcn4 co-ordinates morphogenetic and metabolic responses to amino acid starvation in Candida albicans. EMBO J 21: 5448-5456. doi:10.1093/emboj/cdf507. PubMed: 12374745. 12374745 PMC129063

[19] Chen DC, Yang BC, Kuo TT (1992) One-step transformation of yeast in stationary phase. Curr Genet 21: 83-84. doi:10.1007/BF00318659. PubMed: 1735128. 1735128

[20] Dignard D, Whiteway M (2006) SST2, a regulator of G-protein signaling for the Candida albicans mating response pathway. Eukaryot Cell 5: 192-202. doi:10.1128/EC.5.1.192-202.2006. PubMed: 16400182. 16400182 PMC1360253

[21] Wilson RB, Davis D, Mitchell AP (1999) Rapid hypothesis testing with Candida albicans through gene disruption with short homology regions. J Bacteriol 181: 1868-1874. PubMed: 10074081. 10074081 10.1128/jb.181.6.1868-1874.1999 PMC93587

[22] Reuss O, Vik A, Kolter R, Morschhäuser J (2004) The SAT1 flipper, an optimized tool for gene disruption in Candida albicans. Gene 341: 119-127. doi:10.1016/j.gene.2004.06.021. PubMed: 15474295. 15474295

[23] Askew C, Sellam A, Epp E, Hogues H, Mullick A et al. (2009) Transcriptional regulation of carbohydrate metabolism in the human pathogen Candida albicans. PLOS Pathog 5: e1000612 PubMed: 19816560. 19816560 10.1371/journal.ppat.1000612 PMC2749448

[24] Nantel A, Rigby T, Hogues H, Whiteway M (2006) Microarrays for studying pathology in Candida albicans. In: Kavanaugh K Hoboken NJ: Wiley Press.

[25] Jeffries TW, Van Vleet JR (2009) Pichia stipitis genomics, transcriptomics, and gene clusters. FEMS Yeast Res 9: 793-807. doi:10.1111/j.1567-1364.2009.00525.x. PubMed: 19659741. 19659741 PMC2784038

[26] Wohlbach DJ, Kuo A, Sato TK, Potts KM, Salamov AA et al. (2011) Comparative genomics of xylose-fermenting fungi for enhanced biofuel production. Proc Natl Acad Sci U S A 108: 13212-13217. doi:10.1073/pnas.1103039108. PubMed: 21788494. 21788494 PMC3156214

[27] Leandro MJ, Fonseca C, Gonçalves P (2009) Hexose and pentose transport in ascomycetous yeasts: an overview. FEMS Yeast Res 9: 511-525. doi:10.1111/j.1567-1364.2009.00509.x. PubMed: 19459982. 19459982

[28] Noble SM, Johnson AD (2005) Strains and strategies for large-scale gene deletion studies of the diploid human fungal pathogen Candida albicans. Eukaryot Cell 4: 298-309. doi:10.1128/EC.4.2.298-309.2005. PubMed: 15701792. 15701792 PMC549318

[29] Schwartz K, Wenger JW, Dunn B, Sherlock G (2012) APJ1 and GRE3 homologs work in concert to allow growth in xylose in a natural Saccharomyces sensu stricto hybrid yeast. Genetics 191: 621-632. doi:10.1534/genetics.112.140053. PubMed: 22426884. 22426884 PMC3374322

[30] Wenger JW, Schwartz K, Sherlock G (2010) Bulk segregant analysis by high-throughput sequencing reveals a novel xylose utilization gene from Saccharomyces cerevisiae. PLOS Genet 6: e1000942 PubMed: 20485559. 20485559 10.1371/journal.pgen.1000942 PMC2869308

[31] Jeffries TW, Grigoriev IV, Grimwood J, Laplaza JM, Aerts A et al. (2007) Genome sequence of the lignocellulose-bioconverting and xylose-fermenting yeast Pichia stipitis. Nat Biotechnol 25: 319-326. doi:10.1038/nbt1290. PubMed: 17334359. 17334359

[32] Liang L, Zhang J, Lin Z (2007) Altering coenzyme specificity of Pichia stipitis xylose reductase by the semi-rational approach CASTing. Microb Cell Factories 6: 36. doi:10.1186/1475-2859-6-36. PubMed: 18028553. PMC2213684 18028553

[33] Watanabe S, Abu Saleh A, Pack SP, Annaluru N, Kodaki T et al. (2007) Ethanol production from xylose by recombinant Saccharomyces cerevisiae expressing protein-engineered NADH-preferring xylose reductase from Pichia stipitis. Microbiology 153: 3044-3054. doi:10.1099/mic.0.2007/007856-0. PubMed: 17768247. 17768247

[34] Watanabe S, Pack SP, Saleh AA, Annaluru N, Kodaki T et al. (2007) The positive effect of the decreased NADPH-preferring activity of xylose reductase from Pichia stipitis on ethanol production using xylose-fermenting recombinant Saccharomyces cerevisiae. Biosci Biotechnol Biochem 71: 1365-1369. doi:10.1271/bbb.70104. PubMed: 17485825. 17485825

[35] Walfridsson M, Bao X, Anderlund M, Lilius G, Bülow L et al. (1996) Ethanolic fermentation of xylose with Saccharomyces cerevisiae harboring the Thermus thermophilus xylA gene, which expresses an active xylose (glucose) isomerase. Appl Environ Microbiol 62: 4648-4651. PubMed: 8953736. 8953736 10.1128/aem.62.12.4648-4651.1996 PMC168291

[36] Brat D, Boles E, Wiedemann B (2009) Functional expression of a bacterial xylose isomerase in Saccharomyces cerevisiae. Appl Environ Microbiol 75: 2304-2311. doi:10.1128/AEM.02522-08. PubMed: 19218403. 19218403 PMC2675233

[37] Gillum AM, Tsay EY, Kirsch DR (1984) Isolation of the Candida albicans gene for orotidine-5'-phosphate decarboxylase by complementation of S. cerevisiae ura3 and E. coli pyrF mutations. Molecular General Genet MGG 198: 179-182. doi:10.1007/BF00328721. 6394964

[38] Brachmann CB, Davies A, Cost GJ, Caputo E, Li J et al. (1998) Designer deletion strains derived from Saccharomyces cerevisiae S288C: a useful set of strains and plasmids for PCR-mediated gene disruption and other applications. Yeast 14: 115-132. doi:10.1002/(SICI)1097-0061(19980130)14:2. PubMed: 9483801. 9483801

