# Supplementary material for: Comparative Xylose Metabolism among the Ascomycetes C. albicans, S. stipitis and S. cerevisiae
Source: PLoS One. 2013 Nov 13;8(11):e80733. doi: 10.1371/journal.pone.0080733 (PMC3827475; doi:10.1371/journal.pone.0080733)
Supplement: Figure S1 — Alignments of A) xylose reductases and B) xylose dehydrogenases of C. albicans, S. stipitis and S. cerevisiae. (DOCX) [file pone.0080733.s001.docx]

A)


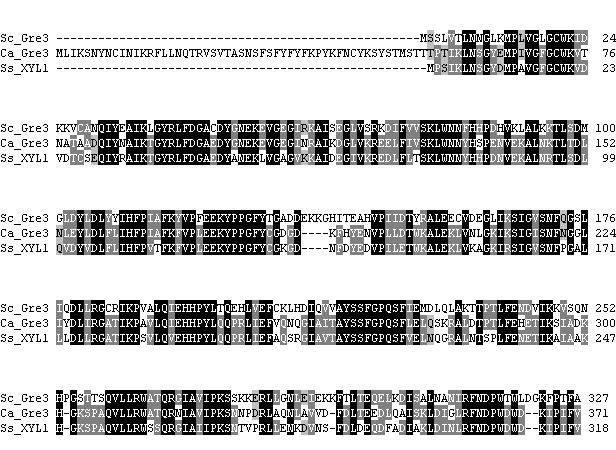


B)


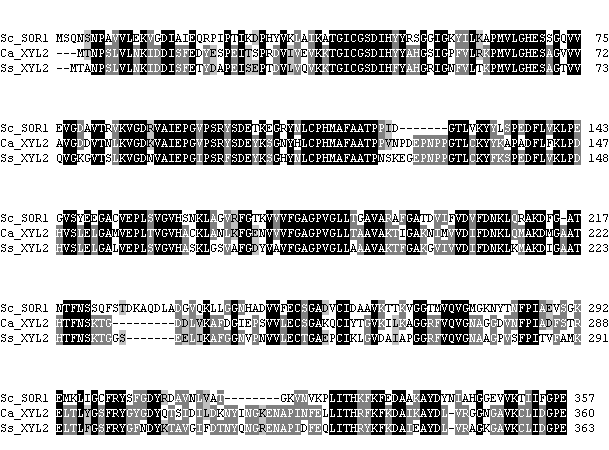


**Figure_S1**. Alignments of A) xylose reductases and B) xylose dehydrogenases of *C. albicans, S. stipitis* and *S. cerevisiae.*

somerases
