# Supplementary material for: Comparative Xylose Metabolism among the Ascomycetes C. albicans, S. stipitis and S. cerevisiae
Source: PLoS One. 2013 Nov 13;8(11):e80733. doi: 10.1371/journal.pone.0080733 (PMC3827475; doi:10.1371/journal.pone.0080733)
Supplement: Figure S3 — Growth analysis of C. albicans wild type (SC5314) and deletion mutants for xylose reductase (gre3-9) and xylitol dehydrogenase (xyl2-16) in synthetic medium (SC) with 2% xylose (SX) or 2% xylitol (ST). Strains were grown aerobically at 30°C, and the optical densities were measured over a period of 4 days. (DOCX) [file pone.0080733.s003.docx]

A)

B)

**Figure_S3.** Growth analysis of *C. albicans* wildtype (SC5314) and deletion mutants for xylose reductase (gre3-9) and xylitol dehydrogenase (xyl2-16) in synthetic medium (SC) with 2% xylose (SX) or 2% xylitol (ST). Strains were grown aerobically at 30°C, and the optical densities were measured over a period of 4 days.
