# Supplementary material for: Comparative Xylose Metabolism among the Ascomycetes C. albicans, S. stipitis and S. cerevisiae
Source: PLoS One. 2013 Nov 13;8(11):e80733. doi: 10.1371/journal.pone.0080733 (PMC3827475; doi:10.1371/journal.pone.0080733)
Supplement: Figure S4 — Alignment of fungal and bacterial xylose isomerases. (DOCX) [file pone.0080733.s004.docx]

**Figure_S4.** Alignment of fungal and bacterial xylose isomerases
